# The New Eclectic, *a Monthly Magazine of Select Literature*

**Published:** 1868-02

**Authors:** 


### BIBLIOGRAPHICAL NOTICE.

THE NEW ECLECTIC, *A Monthly Magazine of Select Literature.*—New York and Baltimore: Lawrence Turnbull and Fridge Murdoch.

We have received the first and second numbers of this new candidate for public favor. It is a substantial octavo, issued monthly, handsomely printed and well gotten up in every particular. Four numbers make a volume of over five hundred pages, making three volumes a year. The selections are made from a wide range of English Literature and evince no little taste and judgment. We heartily commend it to those of our readers who desire to keep up with the current literature of the day.

